# Eccrine Porocarcinoma: A Review of the Literature

**DOI:** 10.3390/diagnostics13081431

**Published:** 2023-04-16

**Authors:** Aikaterini Tsiogka, Dimitra Koumaki, Maria Kyriazopoulou, Konstantinos Liopyris, Alexander Stratigos, Stamatios Gregoriou

**Affiliations:** 1 1st Department of Dermatology-Venereology, Faculty of Medicine, National and Kapodistrian University of Athens, Andreas Sygros Hospital, 16121 Athens, Greece; konstantinosliopyris@gmail.com (K.L.); alstrat2@gmail.com (A.S.); stamgreg@yahoo.gr (S.G.); 2 Department of Dermatology, University Hospital of Heraklion, 71500 Heraklion, Greece; dkoumaki@yahoo.gr; 3 Department of Dermatology and Venereology, 401 General Military Hospital of Athens, 11525 Athens, Greece; mckyriazopoulou@gmail.com

**Keywords:** porocarcinoma, adnexal tumor, skin appendages, rare cutaneous tumors

## Abstract

Eccrine porocarcinoma (EPC) constitutes a rare malignant adnexal tumor, which accounts for about 0.005–0.01% of all cutaneous malignancies. It may develop de novo or arise from an eccrine poroma, after a latency period of years or even decades. Accumulating data suggest that specific oncogenic drivers and signaling pathways may be implicated in its tumorigenesis, while recent data have demonstrated a high overall mutation rate attributed to UV exposure. Diagnosis may be challenging and should rely on the combination of clinical, dermoscopical, histopathological and immunohistochemical findings. The literature is controversial regarding tumor behavior and prognosis and, therefore, there is no consensus on its surgical management, utility of lymph-node biopsy and further adjuvant or systemic treatment. However, recent advances in tumorigenesis of EPC may aid in the development of novel treatment strategies, which could improve survival of advanced or metastatic disease, such as immunotherapy. This review presents an update of the epidemiology, pathogenesis and clinical presentation of EPC and summarizes current data on diagnostic evaluation and management of this rare cutaneous malignancy.

## 1. Introduction

Adnexal malignant tumors of the eccrine sweat glands comprise an heterogenous group of rare cutaneous malignancies, which are generally considered fairly indolent, even though cases of aggressive disease course have been reported in the literature. Among them, eccrine porocarcinoma (EPC) constitutes the most common malignant adnexal tumor, which originates from the intraepidermal section of the excretory duct of the eccrine sweat gland, i.e., the acrosyringium, and accounts for about 0.005–0.01% of all cutaneous malignancies [1]. It was firstly described as “epidermotropic eccrine carcinoma” by Pinkus and Mehregan in 1963, while a few years later, Mishima and Morioka addressed the term “eccrine porocarcinoma”, which is now most frequently used and was included in the 2018 World Health Organization (WHO) classification of skin tumors [2,3,4]. This classification is based on clinical, histopathological and molecular genetic features of each appendageal carcinoma, classifying them into three types: apocrine-eccrine, follicular and sebaceous [2]. Other terms reported so far in the literature are “malignant eccrine poroma” or “malignant hidroacanthoma simplex”. This review presents an update of the epidemiology, pathogenesis and clinical presentation of EPC and summarizes current data on the diagnostic evaluation and management of this rare cutaneous malignancy.

## 2. Materials and Methods

We performed a comprehensive review of the medical literature on EPC using the MEDLINE database via PubMed from inception until February 2023. The literature search involved all fields including title, abstract, keywords and full text, and identified all article types utilizing the terms “eccrine porocarcinoma”, “malignant eccrine poroma” and “malignant hidroacanthoma simplex”. Reference lists of the retrieved articles were manually reviewed to identify any additional relevant articles. Data extraction was independently performed by two reviewers (A.T., S.G.) and included data regarding the epidemiology, pathogenesis, clinical presentation, diagnosis, therapy and prognosis of EPC. The study was exempt from ethics committee approval, since data were retrieved from the published literature.

## 3. Epidemiology

Due to the rarity of EPC, current epidemiological data are mainly derived from a few population-based as well as retrospective studies and meta-analyses. EPC has been shown to mostly affect the elderly population. Systematic reviews of 453, 206 and 120 cases have demonstrated a mean age of presentation ranging from 63.6 to 65.6 years [1,5,6]. Similarly, analysis of the U.S. National Cancer Database from 2004 to 2016 identified 611 cases of EPC with a mean age of presentation of 66 years [7].

In 2010, Blake et al. utilized the Surveillance, Epidemiology and End Results (SEER) Program of the National Cancer Institute to assess the incidence patterns of the cutaneous appendageal carcinoma in the United States (US) between 2001 and 2005 (*n* = 1801). The total incidence rate (IR) of EPC between 2001 and 2005 was estimated at 0.04 per 100,000 person-years, showing a predominance of male gender (IR 0.05 and 0.02 per 100,000 person-years for men and women, respectively) [8]. A following SEER analysis of cutaneous adnexal tumors in the U.S. between 2000 and 2018 (*n* = 9646) reported an age-adjusted IR of 0.045 per 100,000 person-years for EPC, while a retrospective study in Minessota from 2000 to 2010 revealed an overall age- and sex-adjusted IR of 0.2 per 100,000 person-years for both genders [9,10]. A further study, estimating the IR of EPC from the Finnish Cancer Registry from 2007 to 2017 (*n* = 69), provided comparable results, showing an age-adjusted IR of 0.06 and 0.04 per 100,000 person-years for men and women, respectively [11]. Similarly, the IR of EPC was estimated at 0.04 and 0.03 per 100,000 person-years for men and women, respectively, in a population-based study on skin adnexal carcinoma, conducted between 1989 and 2010 in the Netherlands (*n* = 2220) [12]. Significantly higher IR (overall IR 1.9 per person-years; 1.3 and 2.4 per 100,000 person-years for men and women, respectively) was estimated in a study on EPC (*n* = 152), conducted in the east of England between 2004 and 2014 [13]. Overall, despite the discrepancies in the results of the aforementioned studies, the IR of EPC seems to be approximately 0.02–0.2 per 100,000 person-years, with no consistent differences between the two sexes.

## 4. Pathogenesis—Risk Factors

The pathogenesis of EPC is not fully understood. It may develop de novo or arise from its benign counterpart, eccrine poroma, after a latency period of years or even decades [14]. This has been supported by published case series with long-term follow up, as well as the results of a clinicopathologic study of 69 cases reporting that 18% of EPCs demonstrated adjacent features of benign poroma [15,16]. Moreover, the presence of shared oncogenic gene fusions, i.e., hybrid genes formed by the translocation, inversion or deletion of two independent genes, has been observed in both EPC and eccrine poromas, accounting for the production of proteins with oncogenic functions [17,18,19]. Rarely, eccrine poromas may arise within nevus sebaceous and they should be considered in the differential diagnosis of secondary tumors developing on sebaceous nevi [20]. Further reported risk factors comprise chronic ultraviolet (UV) exposure, as well as immunosuppression, while the occurrence of EPC in pre-damaged skin (radiotherapy, trauma) has also been described [21,22].

Although the precise mechanisms behind tumorigenesis in EPC are yet to be elucidated, published studies support the involvement of implicating factors, such as specific oncogenic drivers, including epidermal growth factor receptor (EGFR), tumor protein p53 (TP53) and cyclin-dependent kinase inhibitor 2A (CDKN2A), as well as signaling pathways, including the mitogen-activated protein kinase (MAPK) pathway, on EPC development [23,24,25]. Merilainen et al. investigated the presence of Merkel cell polyoma virus (MCPyV) in eccrine poromas and EPC by means of immunohistochemistry and quantitative polymerase chain reaction (PCR), to evaluate if MCPyV infection may drive the oncogenesis of EPC, as in MCC. The results indicated that MCPyV may not serve as an oncogenic driver for EPC [26]. In another study, Puttonen et al. investigated the distribution and significance of tumor infiltrating lymphocytes (TILs) in the EPC microenvironment, assuming that an altered immunological environment could explain the involvement of chronic UV radiation in the tumorigenesis of EPC. Unlike the known association of TILs with positive prognosis in various skin tumors, such as melanoma and MCC, the authors observed a negative correlation between UV damage and TIL density in EPCs, supporting the immunosuppressive effect of UV radiation on the microenvironment of these tumors [27].

To further elucidate the EPC biology, Denisova et al. investigated the mutational landscape of EPC by performing whole-exome sequencing on 14 EPCs and matched healthy surrounding tissue. The authors observed a high overall median mutation rate attributed to UV exposure, with the most common mutation being the TP53, a cell cycle regulator and tumor suppressor gene, suggesting that inactivation of the p53 pathway plays a significant role in both tumorigenesis and tumor progression. Moreover, the identified mutational processes were found to be comparable to those observed in melanoma, BCC and SCC, suggesting common mechanisms of tumorigenesis [28]. In agreement with this study, Westphal et al. assessed molecular pathway alterations in EPC and revealed high overall mutational burden, mostly attributable to UV-induced mutational signatures, while they found altered protein expression of p53, as well as significant expression of epidermal growth factor receptor (EGFR) protein and programmed death-ligand-1 (PD-L1) [29]. The functional relevance of these findings was supported by reports of EPCs responding to anti-PD-1 and anti-EGFR therapy, suggesting that these pathways may serve as oncogenic drivers in EPC [30,31].

## 5. Diagnosis

Diagnosis of EPC is challenging, as it is characterized by variable and non-specific clinical and histopathological findings, leading to diagnostic delay in most cases. Interestingly, the mean interval between tumor development and diagnosis has been reported to be five to nine years, but it may vary from days to even 60 years, according to the published literature [5,15,16,32,33]. Clinical differential diagnoses comprise benign or malignant lesions, such as pyogenic granuloma, seborrheic keratosis, squamous cell carcinoma (SCC), basal cell carcinoma (BCC), Bowen’s disease, Merkel cell carcinoma (MCC), Paget disease, lymphoma, hidradenoma, etc. Overall, diagnosis should be based on the combination of clinical, dermoscopical, histopathological and immunohistochemical findings.

### 5.1. Clinical Presentation

**Figure 1:**
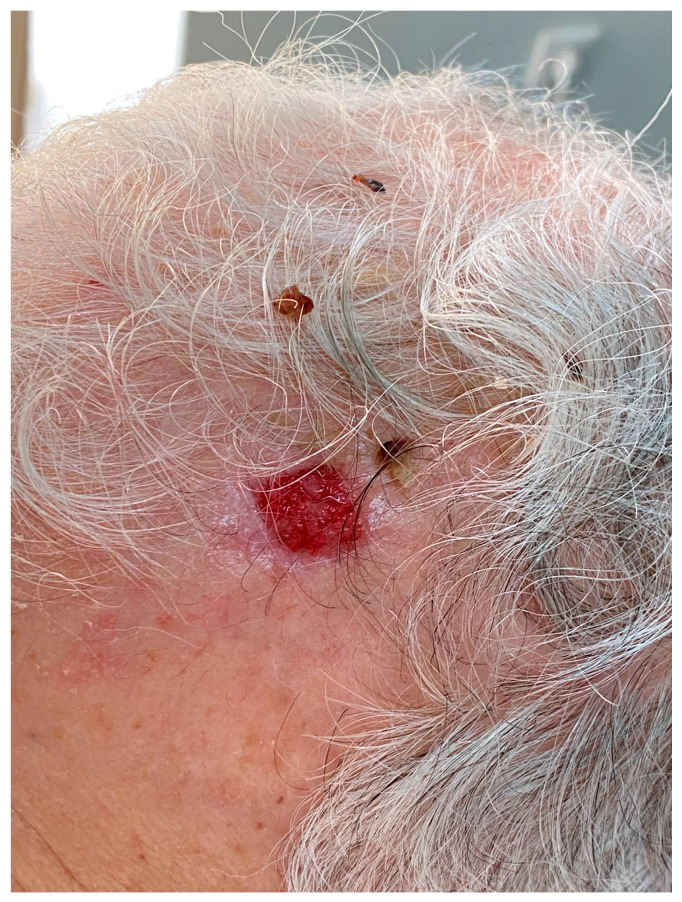
Clinical presentation of eccrine porocarcinoma in a 75-year-old patient, showing an ulcerated lesion of 15 mm in diameter on the left temporal area.

The clinical presentation of EPC is highly variable. Usually, it manifests as an erythematous, violaceous nodular lesion or, more rarely, as a polypoid plaque of violet or erythematous color, growing over weeks to months (Figure 1). It may be asymptomatic or present with itching, ulceration and spontaneous bleeding. The latter should be clinically regarded as signs of malignant transformation, and it has been found to represent a significantly worsening prognostic factor [6,14,34]. The tumor size at time of diagnosis has been reported to range from 1–130 mm, having a mean diameter of 23.88 mm [7]. Behbahani et al. sought to correlate the tumor stage with the disease outcome. Except for the strong association of metastatic disease with worse prognosis, a larger tumor size was also independently associated with decreased overall survival [7]. This also applies to non-melanoma and non-Merkel cell skin cancers, in which a tumor diameter greater than two centimeters is correlated with worse prognosis according to the guidelines of the American Joint Committee on Cancer (AJCC) [35].

In general, predilection sites of EPC are the head and neck area, as well as the lower extremities, followed by the trunk and upper extremities. In a study of 69 cases, as well as a SEER-analysis of 563 cases, the lower extremities were found to be the most commonly affected body site (33.7–44%), followed by the head and neck (18–30.6%) and trunk (19.5–24%) [15,36]. On the contrary, in a Korean study of 37 patients, EPCs were most frequently observed in the head and neck (29.7%) area, aligning with the results of a meta-analysis of 453 patients, in which the most commonly affected locations were the head and neck (39.9%), followed by the lower extremities (33.9%), the upper extremities (8.8%), the back (5.1%) and chest (4.6%) [5,37]. Development of EPC has been also reported on scars, sites of irradiation, lymphedema or further unusual localizations, such as the genital or perianal region [5,22,38].

### 5.2. Imaging

**Figure 2:**
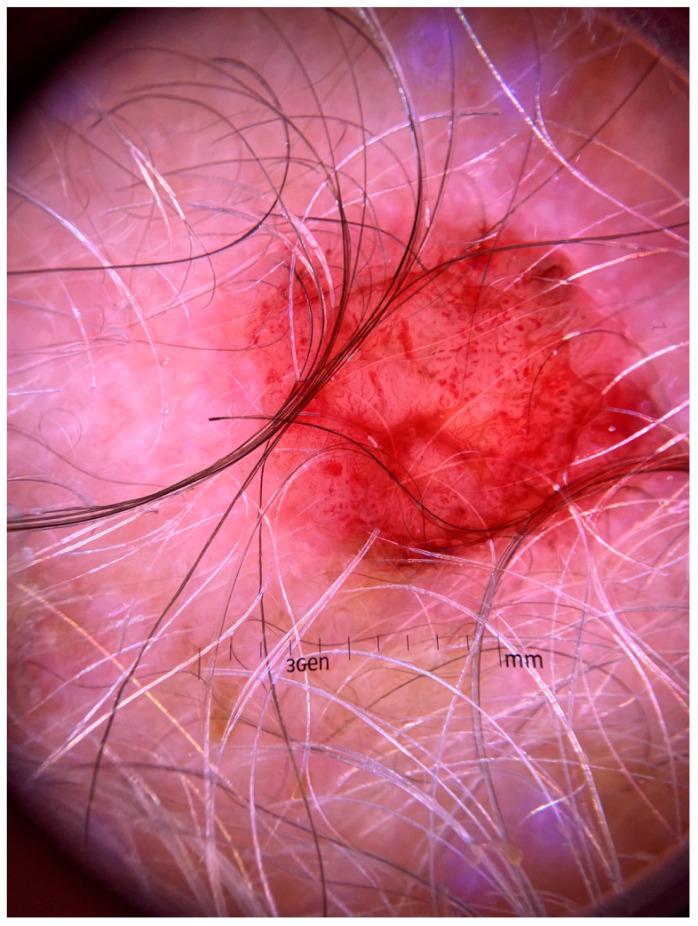
Dermoscopic picture of eccrine porocarcinoma exhibiting an atypical vascular pattern with polymorphous vessels and pink-whitish ovoid areas.

There have only been a few reports addressing the dermoscopic findings of EPC. An atypical vascular pattern with a diffuse arrangement of focused and unfocused polymorphous vessels as well, as pink-whitish ovoid areas, constitute the most commonly reported features (Figure 2) [39]. However, cases exhibiting fine scaling and erosions, as well as structureless pink-whitish areas with no apparent vasculature, have also been described [40]. Pinheiro et al. provided reflectance confocal microscopy (RCM) findings of a relapsing EPC. Aggregates of poroid cells clustered in nests, surrounded by elongated and tortous canalicular vessels and a dark stroma were observed. In addition, roundish dark structures corresponding to areas of ductal differentiation within the nests were also seen [39]. 

Finally, a few studies have addressed the radiological features of EPC. In ultrasound, it has been found to exhibit a multilobulated, well-defined lesion within the dermis and subcutaneous tissue, showing hypoechoic and heterogenous solid components with increased peripheral vascularity, as well as cystic components containing echoic spots [41]. Finally, in magnet resonance imaging (MRI), EPC has been described to include a pedunculated configuration, a homogenous T2 signal, hyperintensity in T1 weighted images and cystic components [41].

### 5.3. Histopathology

The histopathological characteristics of EPC in hematoxylin and eosin staining are diverse and may pose difficulties in the histopathological differential diagnosis of EPC, mainly from SCC. In most cases, large poromatous basaloid epithelial cells exhibiting ductal differentiation and cytologic atypias are observed [33]. This cytologic pleomorphism, along with the increased mitotic activity, tumor necrosis and infiltrative growth pattern, comprise histopathological markers, which enable the differentiation of EPC from benign eccrine poroma. Interestingly, EPC cells may exhibit squamous cell, clear cell and spindle cell differentiation or even melanocyte colonization [42]. In a meta-analysis of 120 EPCs, 25% and 23.4% of cases showed squamous and clear cell differentiation, respectively, while in another study of 33 cases, squamous cell differentiation was observed in 42% and melanocyte colonization in 21% of EPCs [6,33].

**Figure 3:**
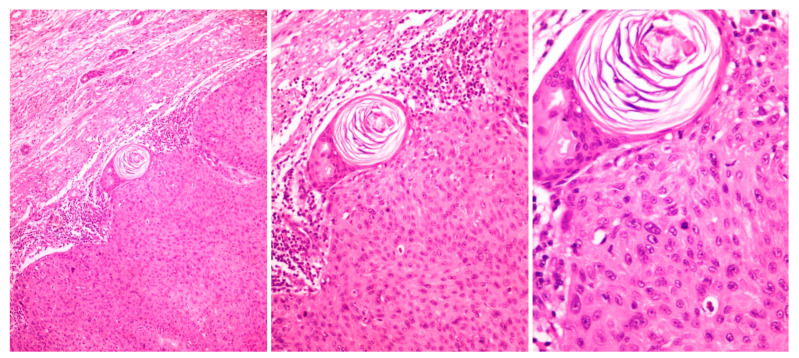
Histopathological picture of eccrine porocarcinoma exhibiting poromatous basaloid epithelial cells and duct formation (hematoxylin and eosin staining, 40×, 100×, 200×).

Duct formation constitutes another significant indicator for the diagnosis of EPC and differentiation from SCC. Histopathological studies have shown that the majority of cases exhibit mature ducts, characterized by cuboidal epithelial cells with an eosinophilic cuticle, even though poorly differentiated EPCs may only show less-developed ducts, which should not be overlooked [15,37]. Further histopathological findings that have been observed in EPC cases are the comedonecrosis; i.e., tumor cells containing central necrosis with an inflammatory reaction similar to a comedone, and a bowenoid pattern, characterized by pleomorphic cells and some multinucleated monster cells (Figure 3) [33,37].

Some studies have proposed the differentiation of EPCs into “pushing”, infiltrative” and “pagetoid”, according to the pattern of the margins of the primary tumor, serving as predictive markers of recurrence [15,32]. In particular, an “infiltrative” pattern with poorly defined margins and malignant clusters into the dermis could be associated with significant higher risk of recurrence than the “pushing” variants, while the “pagetoid” pattern with an intraepidermal spread of tumor cells seems to have an aggressive local potential [32]. Further histopathological features, which are correlated with more aggressive behavior and may guide further therapeutic decisions, comprise a high mitotic rate (>14 per high-power field), a tumor depth > 7 mm and lymphovascular invasion [15].

### 5.4. Immunohistochemistry

Due to the variety of histopathological characteristics, immunohistochemistry may aid in the diagnosis of EPC, even though it has no specific immune profile. Poroid EPC cells typically stain positive for carcinoembryogenic antigen (CEA) and epithelial membrane antigen (EMA), although these markers may also be positive in eccrine poroma, while they also highlight the eccrine ducts of SCC [6,33,43]. On the other hand, CD117 (KIT) and cytokeratin 19 are more likely to be positive in EPC than SCC, aiding in the differential diagnosis among them, as highlighted by two studies on EPCs and SCCs conducted by Goto et al. [44,45]. Although intraepidermal ductal cells are negative for S100, dendritic cells within de-differentiated EPCs and myoepithelial cells of the glandular portions have been found to express S100 [46]. Finally, some studies investigated immunohistochemical expressions of the tumor suppressors p53, Rb and p16, based on emerging data derived from genetic analyses on EPCs. The results suggested that abnormal positive results of any of these three markers could be suggestive for EPC [47]. In cases of pigmented EPC with atypical clinical and dermoscopical characteristics, immunohistochemical markers such as HMB-45, Melan-A and PRAME (Ab219650) may be used towards a differentiation from melanocytic lesions, including melanoma [48,49].

## 6. Disease Course and Prognosis

In general, reports on biologic behavior and prognosis of EPC are variable, given the rarity of the tumor and lack of long-term follow-up data. However, most studies report an approximately 20% potential for local recurrence, 20% for regional lymph node metastasis and 10% for distant metastasis [15,24,50]. The most frequent sites of metastasis are lymph nodes, lungs, liver and brain. In a meta-analysis of 453 EPCs, 31% developed metastasis, with the most common metastatic organ being the lymph nodes (58.5%) followed by the lungs (12.8%) [5]. Robson et al. reported that the presence of lymph node metastasis was associated with 1-year and 3-year overall survival of 88.9% and 39.5%, respectively [15]. Data analysis from the U.S. National Cancer Database registry (*n* = 611) demonstrated an estimated 68.8% 5-year overall survival, which was reduced to 54.3% at 10 years [7]. The same analysis has shown that 8.1% of patients presented with metastatic disease at diagnosis, a finding that was associated with poor prognosis. This percentage has been found to be as high as 31% in further reviews and meta-analyses, while, inversely, recent studies have supported an indolent disease course and low mortality rate, especially in early stages [5,11,51,52]. Despite these discrepancies, most authors agree that early diagnosis and complete surgical removal are the main good prognostic factors [7].

Prognosis of EPC has been reported to be correlated with histopathological characteristics, including the growth pattern, tumor thickness, mitotic rate and lymphovascular invasion, as described above. In particular, a study of Belin et al. demonstrated that the recurrence risk was significantly lower in “pushing” EPCs than in “infiltrative” variants. Moreover, the authors investigated the EGFR expression in EPCs, revealing no correlation between the presence and/or intensity of immunostaining with the risk of recurrence [32]. 

## 7. Treatment

Currently, there is limited evidence regarding the management of EPC and most data may be derived from case series, retrospective studies and meta-analyses. A 10-year retrospective review of 75 cases across the United Kingdom demonstrated great variation in regards to baseline radiological staging, histological reporting and management [53]. Factors which may guide decision making are the histopathological features of the primary tumor, the presence of nodal or distant metastasis as well as patient performance status and comorbidities. 

### 7.1. Tumor Excision

Complete surgical excision should be performed in resectable cases in order to achieve local control of the disease. According to the literature, wide local excision (WLE) with at least 2-mm safety margins constitutes the most commonly applied procedure associated with low recurrence rates and increased survival, as also demonstrated by a meta-analysis of 120 cases of head and neck EPCs, showing that the lack of WLE or Mohs micrographic surgery (MMS) was associated with worse prognosis and decreased overall survival (*p* < 0.001) [6]. Comparison of these treatment modalities revealed a statistical significance regarding recurrence rates (25.3% vs. 0.0% for WLE and MMS, respectively), although this result should be evaluated with caution due to the lack of randomization between the two surgical procedures [6].

Considering the increasing application of MMS, Tolkachjov et al. conducted a retrospective study of patients with EPC treated with MMS at the Mayo Clinic between 1995 and 2013 (*n* = 9). During the mean follow-up period of 3.3 years, no tumors recurred or metastasized [54]. In contrast, a SEER-analysis of 7591 patients with rare malignant adnexal carcinomas, including 644 EPCs, demonstrated no significant differences between the different types of surgical treatment, i.e., excisional biopsy, excision with <1 cm margin and WLE, on survival metrics, as also reported in a previous SEER-17 analysis of 3925 patients with sweat gland neoplasms [51,52]. Whilst consensus regarding the most effective surgical procedure and safety margins is yet to be reached, Berlin et al. proposed that the decision should be determined based on the growth pattern of the primary tumor, with “pushing” EPCs being treated with limited margins (3 mm) and “infiltrative” or “pagetoid” with WLE or MMS with 5-mm margins [32].

### 7.2. Sentinel Lymph Node Biopsy

To date, no formal criteria for the implementation of sentinel lymph node (SLN) biopsy exist. Some authors have proposed that it may be considered in patients with EPC without clinically evident lymphadenopathy, presenting with poor prognostic histopathological signs, including a tumor depth > 7 mm, lymphovascular invasion and >14 mitoses per high-power field [15,33,37,55]. In 2019, Tsunoda et al. conducted a study to specifically assess the utility of SLN biopsy in 13 patients with EPC. Among them, eight underwent SLN biopsy, with only one WLE due to low performance status and four received regional lymph node dissection due to clinical and/or radiological evidence of lymph node involvement [56]. The positivity rate of SLN metastasis was found to be 37.5%, which is higher than that reported for malignant melanoma [56,57]. Based on these results and considering that the lymphatic route is thought to be the main initial metastatic pathway of EPCs, the authors suggested that the early detection of lymph node metastasis via SLN biopsy and subsequent lymph node dissection in positive cases could improve the overall survival of these patients [56]. However, in a SEER analysis of patients with adnexal carcinomas, SLN biopsy was only performed in 8.1% of patients, showing no significant difference in survival based on nodal status [51]. Overall, further studies are needed to evaluate the utility and prognostic significance of SLN biopsy in patients with EPCs.

### 7.3. Radiotherapy

There is little evidence on the use of radiotherapy (RT) in the management of EPCs. It has been implemented for recurrent, locally advanced disease and in primary tumors showing perineural invasion, positive surgical margins or high-grade histology [5]. There have also been reports on metastatic EPCs successfully managed with CyberKnife (for regional lymph node metastasis) or chemoradiation (for distant lymph node metastasis) [58,59]. Recently, Fionda et al. conducted a systematic review to assess the available evidence about the post-operative RT of EPC. The 14 included patients received significantly different total doses and fractionation of RT (from 24Gy/12 fractions to 70Gy/35 fractions) [60]. This inhomogeneity was attributed to the diversity of reported tumor sites, as well as the different RT techniques (i.e., cyberknife, electron beams, intensity modulated RT). There were no cases of locoregional relapse, although distant metastases were observed in 42.8% of patients, suggesting that adjuvant, post-operative RT may be effective for local control of the disease [60]. However, the neutral impact of RT on overall survival has been documented in a meta-analysis of 120 cases, as well as a SEER analysis of 203 cases of EPC [6,61].

### 7.4. Systemic Therapy

Various systemic therapeutic regimes, including mono- or poly-chemotherapy, electrochemotherapy, targeted therapy and immunotherapy, have been employed for the treatment of metastatic or recurrent EPCs. Mijamoto et al. summarized 28 cases treated with chemotherapy, where a detailed description of the clinical course was available [55]. Most of them (60%) with advanced EPC received platinum-based agents such as carboplatin and cisplatin with or without 5-fluorouracil, but only 31.3% of them responded (complete response or partial response), suggesting the resistance of EPC to cytotoxic agents, as also demonstrated by further reports [6,55]. Targeted therapy with cetuximab, a monoclonal antibody targeting EGFR, has been reported to be effective in isolated cases according to the published literature. Godillot et al. performed an analysis of EGFR expression on tumor tissue of a patient with metastatic EPC (lymph nodes, lungs, bones) and proceeded to treatment with cetuximab and paclitaxel, achieving a complete response of lesions without recurrence for six months [30].

Considering that hypermutated tumors are sensitive to immunotherapy, some authors have also evaluated its efficacy in EPC. In that direction, there have been reports of EPCs responding to anti-PD-1 therapy with nivolumab with or without prior anti-EGFR therapy with cetuximab, while some reports have demonstrated tumor response to pembrolizumab following progression to radiochemotherapy [30,31,62]. Although current evidence is limited, immunotherapy may represent a promising treatment option for advanced or metastatic EPCs, warranting further investigation.

The study by Denisova et al., which focused on the characterization of the mutational landscape of EPC using whole-exome sequencing, revealed mutations in TP53 in almost half of the reported cases, which resulted in partial inactivation of the p53 pathway. These findings imply that targeted therapies, such as the poly ADP ribose polymerase (PARP) inhibitors, may have a potential therapeutic role in patients with advanced EPC [28].

## 8. Conclusions

ECP is a rare adnexal tumor with an estimated incidence of about 0.02–0.2 per 100,000 person-years, which may develop de novo or arise from a pre-existing eccrine poroma within years or decades. Accumulating data support the involvement of oncogenic drivers and signaling pathways in its development, as well as a high overall mutation burden attributed to UV exposure. Clinically it mostly manifests as a slowly growing, asymptomatic, erythematous plaque or nodule, while ulceration and spontaneous bleeding should raise suspicion towards a malignant transformation. Its diagnostic evaluation may be challenging due to the diversity of clinical and histopathological findings, while immunohistochemistry should ideally be implemented to enhance the diagnostic accuracy. The published literature is controversial regarding tumor behavior and prognosis, and therefore, there is no consensus on its surgical management, the utility of lymph-node biopsy and further adjuvant or systemic treatment. However, recent advances in tumorigenesis of EPC may aid in the development of novel treatment strategies, which could improve survival of advanced or metastatic disease, such as targeted therapy and immunotherapy. Given the rarity of EPC, management of these patients by multidisciplinary teams is advised, while further multicenter studies will be necessary to establish evidence-based treatment guidelines.

## Data Availability

The data that support the findings of this study are openly available in MEDLINE electronic database.
